# Prevalence of Stress and Associated Factors among Regular Students at Debre Birhan Governmental and Nongovernmental Health Science Colleges North Showa Zone, Amhara Region, Ethiopia 2016

**DOI:** 10.1155/2018/7534937

**Published:** 2018-09-02

**Authors:** Ayele Mamo Abebe, Yilma Girma Kebede, Fikir Mengistu

**Affiliations:** ^1^Department of Nursing, Debre Birhan Health Sciences College, P.O. Box 37, Debre Birhan, Amhara, Ethiopia; ^2^Dessie Health Science College, Ethiopia

## Abstract

**Background:**

Stress is very common among medical students across the globe with the prevalence of 80%. In Ethiopia, the prevalence is 47.7% among college students. Unless it is managed early, it leads to deterioration of academic performance and overall dissatisfaction with life and different serious health problems including anxiety, depression, and suicide. The objective of this study is to assess the prevalence of academic related stress among Debre Birhan governmental and nongovernmental health science college regular students 2015/16.

**Method:**

A cross-sectional study was conducted on a sample of 422 health science students selected by stratified proportional random sampling at Debre Birhan governmental and nongovernmental health science colleges in North Shewa zone, Amhara region, Ethiopia in 2016. Data was collected using the Depression Anxiety Stress Scaling (DASS-21). The level of significance of association for multivariable was determined at P value <0.05.

**Result:**

Prevalence of stress among Debre Birhan governmental and nongovernmental health science college regular students is 4.1%. There is a significant association between stress and sex AOR = 8.525 (1.023, 71.077), fear of examination AOR = 5.096 (1.183, 21.96), living in uncomfortable environment AOR = 14.86 (3.84, 57.515), and perceived present illness AOR = .030 (0.003, 0.286). Depression and anxiety were also seen among 19.7% and 23.6%, respectively.

**Conclusion:**

According to this study, the prevalence of stress among governmental and nongovernmental health science college regular students is not high. However, depression and anxiety were found to be higher than stress and they need immediate management plan. Colleges had better prepared simple screening tool and support students to prevent stress before they cause severe mental health problems.

## 1. Introduction

Worldwide, 80% of college students have stress after exam, papers, problem sets, and other assignments [1]. Stress is considered to be a physiological reaction of an organism where diverse defense mechanism comes into play in order to confront a situation which is perceived as threatening or of increased demand [1, 2]. It is also defined as a particular relationship between the individual and the surroundings which is judged by him/her to be threatening or to overwhelm his/her resources and which puts his/her wellbeing at risk [3]. Academic stress is very common among medical students globally causing mental and emotional pressure, tension, or stress that occurs due to the demands of college life and can in turn negatively affect academic performance [4, 5].

Several studies have reported a high incidence of stress disorders among medical students. Stress among college students can be viewed as the bodies' reaction both neurologically and physiologically to adapt to the new condition [6, 7]. In the same way, the cross-sectional studies done in Malaysia showed that 41% of the participants suffered from psychological stress which correlated directly with depressive symptoms and 84% of respondents were in severe stress, particularly with the academic related stressors [8] and [9], respectively. Similarly, in the other studies done in Malaysia and Thai medical schools, the prevalence of stress among medical students was up to 41.9% and 61.4% [10], respectively. A cross-sectional study carried out at College of Medicine, King Saud University, Saudi Arabia, and the proportion of female students who experienced stress was higher (75.7%) than their counterpart males (57%) [11]. Prevalence of stress was reported to be 20.9% in a Nepali medical school [12], 63.8% in a Saudi Arabian [13], and 90% in a Pakistani medical school [11, 14]. Other relevant research revealed that a total of 2.7% of Sweden medical students had made suicidal attempt due to stress [15].

Another study conducted at School of Nursing, Memorial University of Newfoundland, St. John's, Canada, found that the students experienced high stress levels and that they are at risk of having a physical or psychiatric illness [16]. The prevalence of stress was the highest among the first-year students (78.7%), followed by the second-year (70.8%), third-year (68%), fourth-year (43.2%), and fifth-year students (48.3%) in the study done at Jizan University, Kingdom of Saudi Arabia [17]. On the other cross-sectional study in Ireland, the prevalence of stress among medical students is 71.9% [18]. Similarly in Ghana, out of 37 female students whose age of 46% was in range of 18-19 years, 19% 19-20 years, 32% more than 20 years, and 3% less than 18 years, 97% of them have moderate stress and 3% have severe stress. The major factors contributing to stress were environmental factor 40%, intrapersonal factor 30%, academic factor 19%, and interpersonal factor 11%. For 78 % of subjects, the absence of calm and quiet environment and extremes of climate were stress inducing factors. Additionally inadequate water supply and inadequate supply of electricity had contribution to induce stress in 19% and 12%, respectively [19]. Furthermore stress among students results in impaired judgment, absenteeism self-medication, and addiction to substances like khat chewing, smoking cigarettes, and alcohol drinking [20]. Chronic exposure to stressful conditions leads to deterioration of academic performance, loss of memory, poor relationship with peers and family members, and overall dissatisfaction with life [21]. It can also lead to serious health problems like hypertension, heart attack and stroke, diabetes mellitus and obesity, accelerated aging [22], impaired immune system, suppressed fertility, digestive problem, loss of appetite, increased anxiety, and depression that finally leads to suicide [23].

The students also face social, emotional, physical, and family problems which may affect their learning ability and academic performance [24]. In recent years, there is growing appreciation of stressors involved in medical and nursing training college students, especially freshmen, who are a group particularly prone to stress due to the transitional nature of college life [25, 26]. Academic factors were perceived as greater cause of stress [27]. Medical students revealed that most common sources of stress were staying in hostel, high parental expectations, vastness of syllabus, tests/ exams, and lack of time and facilities for students [12, 28].

In cross-sectional study conducted at Jimma University, Ethiopia, the prevalence of stress among nursing students was 47.7%. Similarly in the other studies, the class of the students and their courses were found to be signified with the stress level of nursing students [29–32]. In similar manner, nursing students are therefore physically, emotionally, and intellectually stressed by their competency system [33, 34]. Therefore, the purpose of study was to assess the prevalence and associated factors of stress among regular college students. Assessing the prevalence and associated factors of stress among regular college students is important for stakeholders to take measures.

## 2. Materials and Methods

A cross-sectional study design was conducted on one governmental (Debre Birhan Health Science College) and two nongovernmental health science colleges' (Kea Med and Victory Health Science Colleges) regular students at Debre Birhan town, North Showa zone, Amhara region, Ethiopia. Debre Birhan town is found in Amhara region at a distance of 130 km northeast of Addis Ababa. The study population was regular students (nursing students) in each of the health science colleges in 2015/16.

### 2.1. Sample Size Determination

The minimum number of samples required for this study was determined by using single population proportion formula considering the following assumptions: P = 47.7% (0.477) [28], q = 1-p, d = absolute precision or tolerable margin of error (d) = 5% = 0.05, Z*α*/2 = 1.96 corresponding to 95% confidence level.

(1) n=Zα/22∗pqd21.962×0.4771−0.4770.052=383

The final sample size was 422 including 10% of nonresponse rate.

### 2.2. Sampling Techniques

The participants were selected by stratified random sampling technique from DBHSC, VICTORYHSC AND KEA MEDHSC, so that Debre Birhan Health Science was the first stratum, Victory Health Science College was the second stratum, and Kea Med Health Science College was the third stratum. The calculated sample size was proportionally allocated in each college and then proportionally divided according to their level of stay for each college and the sample was taken independently from each level of the three health science colleges by systematic random sampling. After calculating the interval “k” from each level, the first sampling unit was selected randomly from each level from the first interval of “k”; frame of each stratum means the list of all study population in each stratum by their ID numbers. After listing the study population by their ID numbers in each stratum and by using population proportion to size, each stratum had its own sample size. The study subjects were selected using systematic random sampling. The sampling interval (K) was obtained by dividing each stratum's total number to the sample size of each stratum (Table 1).

### 2.3. Operational Definitions

 
**Stress: **according to DASS-21, if the respondent scores 15 and above.
 
**Depression: **according to DASS-21, if the respondent scores 9 and above.
 
**Anxiety:** according to DASS-21, if the respondent scored 7.

### 2.4. Data Collection Tools and Procedures

Data was collected by interviewing students using semistructured questionnaire that includes tools used to evaluate the prevalence of stress adopted by** Depression Anxiety Stress Scaling (DASS-21)**: each of the three DASS-21 scales contains 7 items divided into subscales with similar contents. The depression scale assessed dysphonia, hopelessness, devaluation of life, self-deprecation, lack of interest or involvement, anhedonia, and inertia. The anxiety scale assessed autonomic arousal, skeletal muscle effect, situational anxiety, and subjective experiences of anxious affect. The stress scale is sensitive to levels of chronic nonspecific arousal. It assessed difficulty in relaxing, nervous arousal, and being easily upset (agitated, irritable), overreactive, and impatient. Score for DASS-21 was calculated by summing the scores for the relevant items and multiplying by 2. The rating scale is as follows: 0 = never applied to me, 1 = applied to me some times, 2 = applied to me often, and 3 = applied to me almost always. This instrument was not patented. Data was collected by six trained clinical nurses for a period of fourteen days.

### 2.5. Data Quality Assurance

The questionnaire was translated to Amharic to be understood by all participants and translated back to English. The Cronbach's alpha value of the translated questionnaire was 0.80. This means the questionnaire was valid and reliable to assess the stress. Two days of training was given for data collectors 8 days before the starting day of data collection. Pretest was given for 22 students before four days of the actual data collection date at Debre Birhan TVET students that will not include in main data. Based on the finding from the pretest, the questionnaire did not need revision. The data collectors were supervised daily and the filled questionnaires were also checked daily by the principal investigator for completeness and consistency.

### 2.6. Data Processing and Analysis

After data has been checked for completeness and consistency, then it was coded and entered in the computer using Epi info version 7 software. Then, it was analyzed by using SPSS version 20. Descriptive statistic was used to explain the study participants in relation to study variables. P value less than 0.25 was considered as statistically significant. Binary logistic regression analysis was conducted to identify associated factors of academic stress.

## 3. Result

### 3.1. Sociodemographic Characteristics

Out of 422 sample participants, 416 completed the interview questionnaire with the response rate of 98.6%. Among the study subjects 39.2% (163) were male. Among the study participants, 320 (76.9%) participants were 18-20 years of age group with mean age 19.8 years (SD±1.5 years). 96.4% (401) were single, and 98.6% (410) were Amhara ethnicity, the rest being Oromo, Tigre, Gurage, and Agewu. Three hundred and eighty-six were orthodox Christian and the rest were Muslim and protestant. Out of the participants 33.7% (140) were payee or governmental college students. In terms of level of education, level II students were 48.3% (201), level III 28.1% (117), and level IV 23.6% (98). All of the participants were living out of the colleges (Table 2).

### 3.2. Prevalence of Stress

The prevalence of stress among Debre Birhan governmental and nongovernmental regular health science college participants is 3.6% and 4.3 %, respectively. The prevalence of stress among male and female is 0.6% and 6.3 %, respectively. Therefore, stress is more common in female than in males in both governmental and nongovernmental health science colleges (Table 3).

### 3.3. Factors Associated with Stress

In bivariate analysis, sex, age, environment, level of education, presence of illness, and fear of exam have shown association with the stress whereas in multivariate analysis, sex, environment, presence of disease, and fear of exam were significantly associated with the stress.

The results of bivariate and multivariate logistic regression analysis for potential risk factor of academic related stress are shown in Table 4 (n=416).

## 4. Discussion

Stress is considered to be a physiological reaction of an organism where various defense mechanisms come into play in order to confront a situation which is perceived as threatening or of increased demand. In this study the prevalence of stress was 4.1%. From this prevalence 3.6% were female. This study was lower than the study done by Shaikh BT et al. at College of Medicine, King Saud University, Saudi Arabia, in which the proportion of female students who experienced stress was higher (75.7%) than their counterpart males (57%) [11]. The possible reason may be due to the environmental and cultural difference between the study areas.

According to this study, sex and stress have been significantly associated on multivariable analysis AOR = 8.525 (1.023, 71.077). The odd of developing stress in female is 8.5 times higher than males. Living in uncomfortable environmental condition has strong association with stress AOR = 14.86 (3.84, 57.515). The odds of developing stress in those who live in uncomfortable environment are 14.8 times higher than those who live in comfortable environment. This study also supported by the study done in Ghana showed that lack of calm and quite environment caused stress for majority of the study participants [19]. Similarly according to the study conducted in Jimma University uncomfortable environment caused stress for 35.7% of the participants [29, 32]. Fear of examination was significantly associated with stress AOR = 5.096 (1.183, 21.955). The odds of developing stress among those who have fear of examination are 5.1 times higher than those who have no fear of examination. There is similarity with the study done at College of Medicine, King Saud University, Riyadh, Saudi Arabia, which shows that it was demonstrated in both laboratory and self-report questionnaires that students report and experience higher levels of anxiety from the objective structured clinical examination (OSCE) than from the written examinations [35–37].

The finding of this study was below when compared with that systematic review published in 2006 in US and Canadian medical students suffering from high incidence of stress than general population [7]. The study conducted in School of Nursing Memorial University of Newfoundland Canada found that students experienced high stress level and they are at risk of having physical or psychiatric illness [8]. This means that this Nepali study was higher than this study maybe due to the envirnmental factors and cultural factors [12].

It is still below when compared with that of the study done in Saudi Arabian medical students with prevalence of 63.8% [10] and study made at the public University in Guadalajara Mexico among 527 students, in which 35.3% of them displayed high level distress, 44.8% medium level of stress, and 19.9% of them low level [1]. The recent result was below the study done on Pakistan medical students in which the prevalence of stress was 90% [11, 14]. A survey conducted on 179 University Kabangsaan Malaysia medical students revealed prevalence of stress being 84% due to academic related problems [9]; it was also below the result of the cross-sectional study done in medical college of Kerala among 96 medical students using different tools like MSSQ, PSS-10, and GHQ-12, and the prevalence of stress was 93.75%, 69.79%, and 65.62%, respectively [8]. In a cross-sectional study done at College of Medicine at King Saud University, Riyadh, female students were 75.7% and male students were 57% stressed [11]. Another cross-sectional study was conducted to determine the prevalence and the factors associated with stress among 385 medical students at Jizan University in Kingdom of Saudi Arabia using GHQ tool. The prevalence of stress among years of study was as follows: first year 78.7%, second year 70.8%, third year 68%, fourth year 43.2%, and fifth year 48.3% [16]. In recent years there is a growing appreciation of stressors involved in medical and nursing training colleges students especially freshmen who are a group particularly prone to stressors [25, 37]. The cross-sectional study was conducted to determine the prevalence and the factors associated with stress among 385 medical students at Jizan University in Kingdom of Saudi Arabia. The prevalence of stress among medical students was 71.9%, with females being more stressed (77%) than the males (64%) [18]. The research done in Belgrade University in Serbia showed that the prevalence of stress was 22-36% [20]. In Malaysia and Thai medical school the prevalence of stress was 41.9% and 61.4%, respectively [10]. According to the study done in Ghana trinity college, on 37 female third-year nursing students, 97% of them were moderately and 3% of them were severely stressed [19]. The stress was caused by academic factors, teaching learning staff, clinical experiences [38], financial problems, and death of the patients [30]; although the degree of stress varies, all of them were stressed. The cross-sectional study conducted among medical students, dental and engineering colleges from the urban area of Sangli district Maharashtra, India, in which out of 1224 respondents 299 (24,4%) experienced stress, 187 (27.7%) were female, and 112 (20.4%) males were stressed [17]. The recent result is below when compared to all previous studies.

The possible reason for this discrepancy could be the study tools used, the study population difference, the difference in level of study, and the living condition (living in the campus and not living in the campus). Similarly the sample size taken was also different which could make the difference. The other possible reason might be that there is no research done on diploma students to compare.

## 5. Study Limitation

- The tool used to assess stress was not sensitive.
- It is also impossible to generalize to the whole college students.
- The other possible limitation could be that the study was not supported with qualitative study for triangulation.

## 6. Conclusions

This research showed that small number of students suffer from stress. The predominant factors associated with stress were sex of participant, living in uncomfortable environment, and fear of examination. Therefore, to avoid the stress, the health education should be given on each case and the route cause should be treated.

*Recommendations*

- The Regional Health Bureau should better strengthen continuous support and follow-up of colleges.
- The colleges should better strengthen continuous assessment to reduce fear of examination.
- The college should establish the stress screening and management programme and should focus on female students.
- Students should prepare themselves for outcome based training from the beginning.
- The researcher should conduct other retrospective cohort studies.

## Figures and Tables

**Table 1:** Sample size distribution in each college.

Name of college	No. of students (Ni)	ni = n/NxNi	Ni	K
DBHSC	280	422/837X280	141	280/141=2
VITORYHSC	490	422/837X490	247	490/247=2
KEAMEDHSC	67	422/837X67	34	67/34=2
TOTAL	837		422	

**Key**: Ni = total no. of students in each college; ni = sample size of each college.

N = total no. of students from all colleges = interval at which the second person was selected.

**Table 2:** Sociodemographic characteristics and some variables of the participants among Debre Birhan governmental and nongovernmental health science colleges, Debre Birhan, 2016 (n=416).

**Variables**	**Count**	**Percent**
**Age year**		
**18-20 **	320	76.9
**21-24**	96	23.1
**Sex**		
**Male**	163	39.2
**Female**	253	60.8
**Ethnicity**		
**Amhara**	410	98.6
**Other**	6	1.4
**Religion**		
**Orthodox**	386	92.8
**Others **	30	7.2
**Marital state**		
**Single**	401	96.4
**All other**	15	3.6
**Payment condition (payee or payer)**		
**Payee (governmental)**	140	33.7
**Payer (nongovernmental)**	276	66.3
**Level of education**		
**Level II**	201	48.3
**Level III**	117	28.1
**Level IV**	98	23.6

**Other ethnicity** = Oromo, Tigre, Gurage, and Agew. **Other religion** = Muslim and protestant.** Other marital state** = married, separated, and divorced.

**Table 3:** Prevalence of stress 2016 Ethiopia (n=416).

Variable	No stress	%	Yes stress	%
Age (18-20)	308	96.2	12	3.8
(21-24)	91	94.8	5	5.2
Level of education				
Level II	192	95.5	9	4.5
Level III	111	94.9	6	5.1
Level IV	96	98	2	2
Sex				
Male	162	99.4	1	0.6
Female	237	93.7	16	6.3
Type of college				
Governmental	135	96.4	5	3.6
Nongovernmental	264	95.7	12	4.3
Total prevalence of stress seen was 4.1%.				

**Table 4:** 

Variable	Stress %		COR (95% CI)	AOR (95%CI)
	Yes%	No%		
Sex				
Male	1	162	1	1
Female	16	237	10.94 (1.44, 83.3)	8.525 (1.023, 71.077)
Age				
18-20	12	308	1	
21-24	5	91	1.410 (.484, 4.108)	
Environment (yes)			1	1
(No )	17	399	13.24 (3.72, 9,47)	14.86 (3.84, 57.515)
Level of education				
Level II	9	192	2.250 (0.477, 10.618)	
Level III	6	111	2.595 (.512, 13.156)	
Level IV	2	96	1	1
Presence of illness (yes)	17	399	0.084 (.011, 0.641)	0.030 (.003, 0.286)
(No)			1	1
Fear of exam	17	399	2.450 (.832, 7.212)	5.096 (1.183, 21.955)
(No)			1	1

## Data Availability

Data supporting the conclusions of this article are available by request to Yilma Girma Kebede and Ayele Mamo Abebe. The relevant raw data will be made available to researchers wishing to use them for noncommercial purposes.

## Abbreviations

AC: Academic commission
DBU: Debre Birhan University
ERB: Ethical Review Board
HSC: Health Science College
HIT: Health information technology
AOR: Adjusted odds ratio
OSCE: Objective structured clinical examination
DASS: Depression Anxiety Stress Scale
SPSS: Statistical Package of Social Science.
